# Patchouli alcohol suppresses *Helicobacter pylori*–associated gastric cancer by activating PXR to inhibit the Wnt/β-catenin pathway and EMT

**DOI:** 10.3389/fphar.2026.1794025

**Published:** 2026-04-22

**Authors:** Xinyu Zhang, Qingling Zeng, Pengpeng Guo, Yifei Xu, Rui Ma, Jingwei Li

**Affiliations:** 1 Institute of Gastroenterology, Shenzhen Traditional Chinese Medicine Hospital, The fourth Clinical Medical College of Guangzhou University of Chinese Medicine, Shenzhen, Guangdong, China; 2 School of Pharmaceutical Sciences, Guangzhou University of Chinese Medicine, Guangzhou, Guangdong, China; 3 The First Clinical Medical College of Guangzhou University of Chinese Medicine, Guangzhou, Guangdong, China; 4 Shenzhen Maternity and Child Healthcare Hospital, Women and Children’s Medical Center, Southern Medical University, Shenzhen, Guangdong, China

**Keywords:** EMT, gastric cancer, *Helicobacter pylori*, Patchouli alcohol, PXR, Wnt/β-catenin signaling

## Abstract

**Background:**

*Helicobacter pylori* (HP) infection is a principal risk factor for gastric cancer, driving tumorigenesis through chronic inflammation, disruption of the epithelial barrier, and genomic instability. Patchouli alcohol (PA), the major bioactive constituent of *Pogostemon cablin*, exhibits anti-inflammatory, antioxidant, and antitumor properties; however, its role and underlying mechanisms in HP-associated gastric cancer remain poorly understood.

**Purpose:**

We hypothesized that HP infection suppresses the activity of the pregnane X receptor (PXR), thereby relieving its inhibitory effect on Wnt/β-catenin signaling and epithelial–mesenchymal transition (EMT), promoting gastric cancer invasiveness. PA was proposed as a PXR agonist capable of counteracting HP-induced malignant phenotypes via modulation of the PXR–Wnt/EMT axis.

**Methods:**

A multi-level integrative approach was employed: (1) single-cell transcriptomic analysis to characterize expression and regulatory features of the PXR–Wnt–EMT axis in HP-positive versus HP-negative gastric cancer epithelial cells; (2) virtual knockout and TCGA-STAD cohort analyses to assess the causal relationship between PXR and Wnt/EMT signaling; (3) molecular docking and 100 ns molecular dynamics simulations to evaluate PA binding to the PXR ligand-binding domain (LBD) and complex stability; (4) metabolomic and proteomic analyses in HP-associated gastric cancer models to validate PA-mediated modulation of metabolic networks and Wnt/EMT signaling.

**Results:**

Single-cell analysis revealed that HP-positive epithelial cells exhibited reduced PXR activity alongside upregulation of Wnt/β-catenin signaling and EMT transcription factors. Virtual PXR knockout recapitulated Wnt/EMT transcriptional activation, and TCGA-STAD analysis confirmed a significant negative correlation between PXR activity and Wnt/EMT signaling. Molecular docking and MD simulations demonstrated stable binding of PA to the PXR LBD. Metabolomic profiling revealed disrupted bile acid and lipid metabolism in HP model mice, partially restored by PA treatment. Proteomic and immunofluorescence analyses showed that PA downregulated Wnt pathway proteins (Dvl3, Tcf7l2) and mesenchymal EMT markers (N-cadherin, MMP9) while upregulating the Wnt inhibitor APC and epithelial marker E-cadherin, collectively reversing HP-induced Wnt/EMT hyperactivation.

**Conclusion:**

PA activates PXR through both direct and indirect mechanisms, thereby suppressing Wnt/β-catenin signaling and EMT, and ultimately inhibiting the initiation and progression of H. pylori–associated gastric cancer. This study elucidates the molecular link between HP-induced PXR downregulation and aberrant Wnt/EMT activation, highlighting PA as a potential therapeutic and preventive candidate for HP-related gastric cancer.

## Introduction

Gastric cancer ranks as the fifth most common malignancy worldwide, with over 1 million new cases and approximately 769,000 deaths annually, and a disproportionately high disease burden in East Asia (Zhang et al., 2024). HP infection is a well-established risk factor and has been classified as a Group I carcinogen by the World Health Organization. Epidemiological studies show that HP infection increases gastric cancer risk by 2–3-fold, with nearly half of the global population harboring the bacterium (Huang et al., 1998). HP drives gastric carcinogenesis through multiple mechanisms, including chronic inflammation, disruption of the mucosal barrier, and genomic instability (Salvatori et al., 2023). Current interventions, however, face major limitations. HP eradication offers limited benefit for patients with established intestinal metaplasia, representing a so-called “point of no return” (Wu et al., 2021), and increasing antibiotic resistance (clarithromycin 20%–30%, metronidazole 40%–50%) further compromises treatment efficacy (Liu et al., 2019). For patients with HP-associated gastric cancer, the 5-year survival rate in advanced stages remains below 10% (Evdokimova et al., 2024). These challenges highlight the urgent need to elucidate HP-mediated molecular mechanisms and identify novel therapeutic targets.

In HP-associated gastric cancer, the Wnt/β-catenin signaling pathway and EMT are closely interconnected and play central roles in tumor progression. Aberrant Wnt activation, characterized by APC inactivation, nuclear β-catenin accumulation, and overexpression of downstream targets, is associated with tumor stemness, chemoresistance, and poor prognosis (Chiurillo, 2015; Shi et al., 2025) EMT, in which epithelial cells acquire mesenchymal traits and migratory capacity, involves downregulation of E-cadherin, upregulation of N-cadherin and Vimentin, and matrix metalloproteinase activation (Kozak et al., 2020; Okubo et al., 2017). Nuclear β-catenin directly regulates EMT transcription factors, including SNAI1/2, ZEB1, and TWIST, thereby promoting invasion and metastasis (Yook et al., 2006; Heuberger and Birchmeier, 2010). Wnt activity correlates positively with EMT scores in gastric cancer, and their synergy predicts patient outcomes (Han et al., 2024). Nevertheless, the mechanisms by which HP infection activates the Wnt/EMT axis remain unclear. Elucidating the key molecular nodes linking HP to Wnt/EMT activation could reveal novel targets for intervention.

The PXR (gene NR1I2), a member of the nuclear receptor superfamily, classically regulates the expression of xenobiotic-metabolizing enzymes (e.g., CYP3A4) and transporters (e.g., MDR1) (Chai et al., 2020; Robbins and Chen, 2014). Emerging evidence indicates that PXR functions extend beyond metabolism, exhibiting tumor type–dependent bidirectional effects in cancer (Robbins and Chen, 2014; Kamaraj et al., 2022). In gastrointestinal tumors, PXR exerts a tumor-suppressive role, inhibiting intestinal inflammation and tumorigenesis through antagonism of NF-κB signaling (Deuring et al., 2019; Cheng et al., 2014). Notably, nuclear receptors functionally oppose the Wnt/β-catenin pathway, as receptor activation can inhibit β-catenin transcriptional activity via competition for co-activators (Lu et al., 2005; Ming et al., 2023), suggesting that PXR may serve as an upstream negative regulator of the Wnt/EMT axis. However, the expression and regulatory role of PXR in HP-associated gastric cancer remain uncharacterized. Given that HP infection induces metabolic dysregulation in the gastric mucosa and that endogenous metabolites, such as bile acids, are natural PXR ligands, we hypothesize that HP may suppress PXR activity, thereby releasing its inhibitory control over the Wnt/EMT axis and promoting invasive gastric cancer progression.

PA, the principal bioactive component of the traditional Chinese medicinal herb *Pogostemon cablin*, is a tricyclic sesquiterpenoid (Junren et al., 2021). Pharmacological studies have demonstrated that PA exhibits diverse bioactivities, including anti-inflammatory, antioxidant, gastroprotective, and antitumor effects (Lian et al., 2018; Song et al., 2021). In gastrointestinal disease models, PA protects the mucosal barrier and suppresses inflammatory responses (Liu et al., 2017). Structurally, sesquiterpenoids can interact with nuclear receptor ligand-binding domains, and certain terpenoids have been reported to modulate PXR activity (Carazo et al., 2019), suggesting that PA may function as a potential PXR ligand. If PA directly activates PXR, it could inhibit HP-associated gastric carcinogenesis via the PXR–Wnt/EMT axis, though this hypothesis remains to be experimentally validated.

Based on this background, we formulated the core scientific hypothesis: HP infection suppresses PXR activity, thereby releasing its negative regulation of the Wnt/β-catenin pathway, activating the EMT program, and promoting an invasive gastric cancer phenotype; PA may serve as a PXR agonist to counteract this pathological process. To test this hypothesis, a multi-level integrative approach was employed: (1) single-cell transcriptomic analysis to characterize differential expression and transcriptional regulatory features of the PXR–Wnt–EMT axis in HP-positive versus HP-negative gastric cancer; (2) integration of virtual knockouts with TCGA cohort analysis to delineate the causal relationship between PXR and the Wnt/EMT axis; (3) molecular docking and molecular dynamics simulations to evaluate the direct binding of PA to PXR; and (4) metabolomic and proteomic analyses to validate PA’s regulatory effects on metabolic networks and the Wnt/EMT pathway in HP-associated gastric cancer models. This study aims to elucidate the molecular mechanism underlying “HP infection → PXR inhibition → Wnt/EMT activation” and provide experimental evidence supporting PA as a potential therapeutic agent for HP-associated gastric cancer.

## Materials and methods

### Single-cell RNA sequencing analysis

Single-cell RNA sequencing (scRNA-seq) data were obtained from the Gene Expression Omnibus (GEO) database (GSE150290 and GSE249874). Samples included pathologically confirmed gastric adenocarcinoma tissues with clearly defined HP infection status. Data processing was performed using Seurat (v5.0) with the following quality control steps: cells with fewer than 200 detected genes, fewer than 1,000 UMI counts, or log10 (gene/UMI) < 0.7 were excluded. Additionally, cells containing >20% mitochondrial genes or >5% hemoglobin genes were removed. Potential doublets were identified using the DoubletFinder package (v2.0.2). Multi-sample integration was performed with Harmony to correct for batch effects.

Subsequently, principal component analysis (PCA), nearest-neighbor graph construction (FindNeighbors), and clustering analysis (FindClusters) were performed sequentially, and two-dimensional visualization was generated using the UMAP algorithm. Cell type annotation was based on classical marker genes combined with automatic annotation by SingleR and manual verification: T cells (CD3D, CD3E), B cells (CD79A, MS4A1), NK cells (GNLY, NKG7), plasma cells (JCHAIN, MZB1), myeloid cells (CD14, LYZ, CD68), mast cells (TPSAB1, CPA3), epithelial cells (EPCAM, KRT18, KRT19), endothelial cells (PECAM1, VWF), and fibroblasts (COL1A1, DCN).

Epithelial cell subsets were further extracted and grouped according to HP infection status. Gene set enrichment analysis (GSEA) was used to evaluate pathway activity differences between HP-positive and HP-negative groups, with the MSigDB Hallmark gene set (h.all.v7.5.symbols.gmt) as the reference. Genes were ranked by log2FoldChange, and analyses were performed using the clusterProfiler R package. Pathways with a false discovery rate (FDR)–adjusted q-value <0.25 and |NES| > 1 were considered significantly enriched.

Gene regulatory networks (GRNs) at the single-cell level were constructed using pySCENIC (Single-Cell rEgulatory Network Inference and Clustering). The analysis workflow included: (1) inferring co-expression relationships between transcription factors and target genes using the GRNBoost2 algorithm; (2) performing cis-regulatory element enrichment analysis with RcisTarget to select regulons with direct regulatory evidence; and (3) calculating regulon activity scores (AUC values) in each cell using the AUCell algorithm. Special attention was given to the regulatory activity of transcription factors such as NR1I2 (PXR), LEF1, SNAI2, and ZEB1. Differences in regulon activity between HP-positive and HP-negative epithelial cells were assessed using the Wilcoxon rank-sum test.

Virtual knockout of NR1I2 (PXR) was performed using the scTenifoldKnk method to simulate the suppression of PXR activity under HP infection conditions. This method identifies downstream responsive genes regulated by NR1I2 by comparing the gene regulatory network structures between the wild-type and virtual knockout states. The differential responsive gene sets were subjected to Gene Ontology (GO) enrichment analysis—including biological processes, molecular functions, and cellular components—and KEGG pathway enrichment analysis, implemented with the clusterProfiler R package, with an adjusted p-value (p.adjust) < 0.05 considered statistically significant.

### TCGA-STAD cohort analysis

RNA-seq expression data and clinical information for the stomach adenocarcinoma (STAD) cohort were downloaded from The Cancer Genome Atlas (TCGA) database. Single-sample gene set enrichment analysis (ssGSEA) was used to calculate PXR activity scores, Wnt/β-catenin pathway scores, and EMT scores for each sample. PXR scores were based on NR1I2 and its classical target genes (CYP3A4, CYP2B6, MDR1); Wnt scores were calculated using the MSigDB HALLMARK_WNT_BETA_CATENIN_SIGNALING gene set; EMT scores were calculated using the HALLMARK_EPITHELIAL_MESENCHYMAL_TRANSITION gene set. Spearman correlation analysis was performed to assess relationships among the scores, and samples were divided into high- and low-PXR activity groups based on the median PXR score for GSEA comparison of pathway differences.

Weighted gene co-expression network analysis (WGCNA) was also performed to identify gene modules associated with PXR expression. The workflow included: (1) selecting the top 5,000–8,000 genes with the highest expression variance; (2) choosing a soft-thresholding power to construct a scale-free network; (3) calculating the adjacency matrix and transforming it into a topological overlap matrix (TOM); (4) performing hierarchical clustering to identify gene modules; and (5) calculating the correlation between each module and NR1I2 expression. Gene Ontology (GO) and KEGG pathway enrichment analyses were conducted for modules showing significant correlations.

### Molecular docking and molecular dynamics simulations

The full-length human PXR structure was obtained from the AlphaFold Protein Structure Database (UniProt ID: O54915). The original ligand and water molecules were removed using PyMOL, hydrogen atoms were added, and energy minimization was performed. The three-dimensional structure of PA was obtained from the PubChem database. Molecular docking was carried out with AutoDock Vina, defining the docking grid around the PXR-LBD ligand-binding pocket. Docking results were ranked by binding energy, and the lowest-energy conformation was selected for subsequent analysis. PyMOL was used to visualize the binding mode and identify key interacting residues.

The optimal docking pose served as the initial structure for 100-ns molecular dynamics (MD) simulations conducted with GROMACS. Simulation parameters were as follows: (1) force field: CHARMM36, with ligand parameters generated via CGenFF; (2) water model: TIP3P; (3) system setup: the protein–ligand complex was solvated in a cubic box with ≥1.0 nm distance from the protein to the box edge, and Na^+^/Cl^−^ ions were added for neutralization; (4) energy minimization: steepest descent algorithm, maximum force <1,000 kJ/mol/nm; (5) equilibration: 100 ps NVT at 300 K followed by 100 ps NPT at 1 bar; (6) production run: 100 ns NPT ensemble with 2 fs timestep, saving frames every 10 ps. Trajectory analyses included root-mean-square deviation (RMSD) to assess protein backbone stability, root-mean-square fluctuation (RMSF) to evaluate residue flexibility, solvent-accessible surface area (SASA) to analyze surface exposure, radius of gyration (Rg) to assess molecular compactness, and Gibbs energy landscape (GEL) analysis based on RMSD and principal component (PC1/PC2) space.

Binding free energy between PA and PXR was estimated using the molecular mechanics/Poisson–Boltzmann surface area (MM/PBSA) method. From the equilibrated portion of the MD trajectory (last 50 ns), frames were extracted every 100 ps (500 frames total). The binding free energy (ΔG_bind) was calculated and decomposed as: ΔG_bind = ΔE_vdW + ΔE_elec + ΔG_polar + ΔG_nonpolar – TΔS, where ΔE_vdW is the van der Waals energy, ΔE_elec is the electrostatic energy, ΔG_polar is the polar solvation energy (Poisson–Boltzmann), and ΔG_nonpolar is the nonpolar solvation energy (derived from SASA). Calculations were performed using gmx_MMPBSA.

### Animal experiments

Male C57BL/6 mice (12–15 g) were purchased from Beijing Vital River Laboratory Animal Technology Co., Ltd. and housed in an SPF-grade animal facility (temperature 22 °C ± 2 °C, humidity 50% ± 10%, 12 h light/dark cycle, *ad libitum* access to food and water). All experiments were approved by the Shenzhen PKU-HKUST Medical Center Animal Ethics Committee (Approval No.: 2022-296) and were strictly conducted in accordance with the guidelines for the care and use of laboratory animals.

The HP-associated gastric cancer mouse model was established using N-methyl-N-nitrosourea (MNU) combined with *Helicobacter pylori* (SS1 strain) infection. HP was cultured on Columbia blood agar plates containing 10% fetal bovine serum under microaerophilic conditions (5% O_2_, 10% CO_2_, 85% N_2_) at 37 °C for 48–72 h. Log-phase bacterial suspensions were collected and adjusted to 1 × 10^8^–1 × 10^9^ CFU/mL. Modeling was performed as follows: mice were first given MNU water (240 ppm, every other day for 4 weeks), followed by 12 h fasting, then orally gavaged with 0.2 mL HP suspension per mouse every other day for 4 doses. Subsequently, mice underwent a tumor progression and drug treatment phase of more than 30 weeks.

Experimental groups were: control group (normal feeding), model group (MNU + HP), PCN group (MNU + HP + PCN, PXR agonist positive control), low-dose PA group (PAL, MNU + HP + PA 10 mg/kg/day), and high-dose PA group (PAH, MNU + HP + PA 20 mg/kg/day). PCN (Pregnenolone-16α-carbonitrile, Sigma-Aldrich) was dissolved in DMSO to prepare a 10 mg/mL stock solution, diluted to 3 mg/mL with Tween-80 and saline, and administered by gavage at 30 mg/kg (0.1 mL/10 g) once daily for 3 weeks. Similarly, PA was dissolved in 1% Tween-80 solution and administered once daily by gavage for 3 weeks. Control mice received an equal volume of the vehicle following the same schedule.

At the end of the experiment, mice were fasted for 12 h, and blood samples were collected via the orbital sinus under isoflurane anesthesia for biochemical analyses. Following blood collection, mice were euthanized by cervical dislocation. Stomach tissues were excised, rinsed with physiological saline, and opened along the greater curvature to document gross lesions. Tissue samples were divided: portions were snap-frozen in liquid nitrogen and stored at −80 °C for proteomic analysis, while the remaining tissues were fixed in 4% paraformaldehyde for histopathological examination.

### Cell experiments

The human gastric cancer cell line AGS was obtained from the Cell Bank of the Chinese Academy of Sciences and maintained in RPMI-1640 medium supplemented with 10% fetal bovine serum and 1% penicillin–streptomycin at 37 °C in a humidified incubator with 5% CO_2_. *Helicobacter pylori* strain SS1 was cultured under microaerophilic conditions as described above. For infection, cells were switched to antibiotic-free medium and co-cultured with *H. pylori* at a multiplicity of infection (MOI) of 50–100 for 6–24 h to establish an *in vitro* infection model.

PA was dissolved in DMSO to generate a 100 mM stock solution and diluted to working concentrations before use. Cells were pretreated with PA (10 or 20 μM) for 2 h prior to *H. pylori* infection. The PXR agonist PCN (10 μM) served as a positive control. Equivalent volumes of DMSO (final concentration <0.1%) were added to the control and model groups. All experiments were performed in triplicate.

### Metabolomics analysis

Metabolites were extracted from mouse gastric tissues by homogenization in a pre-cooled methanol/acetonitrile/water mixture (2:2:1, v/v), followed by 1 h of ice-bath ultrasonication and incubation at −20 °C for 1 h. Samples were centrifuged at 16,000 g and 4 °C for 20 min, and the supernatant was collected for LC–MS analysis. Quality control (QC) samples were prepared by pooling equal volumes of all experimental samples and analyzed alongside each batch for data normalization.LC–MS analysis was performed on a Shimadzu Nexera X2 LC-30AD system coupled with an ACQUITY UPLC HSS T3 column (1.7 μm, 2.1 mm × 100 mm, Waters) and a 5500 QTRAP triple quadrupole mass spectrometer. Metabolites were detected in both positive and negative ion modes with a 5 μL injection volume. The mobile phases were 0.1% formic acid in water (A) and acetonitrile (B), with a column temperature of 40 °C and a flow rate of 200 μL/min. Widely targeted metabolites were quantified using multiple reaction monitoring (MRM), and mass spectrometry parameters followed previously published protocols (Cao et al., 2020; Zheng et al., 2020). Raw data were processed using MultiQuant 3.0.2, and peak areas were used for quantification.

### Proteomics analysis

Proteins from tissues or plasma were extracted using SDT lysis buffer (4% SDS, 100 mM DTT, 100 mM Tris-HCl, pH 8.0) or 8 M urea with 100 mM ammonium bicarbonate. Samples were boiled or denatured, subjected to ultrasonication, and centrifuged at 16,000 g to remove insoluble debris. Protein concentrations were determined using the BCA assay.Protein digestion was performed using the FASP method: cysteine residues were reduced with DTT and alkylated with IAA in UA buffer, followed by overnight trypsin digestion (enzyme:protein ratio 1:50) at 37 °C. Resulting peptides were desalted using C18 StageTips, and peptide concentrations were measured by OD280.LC–MS/MS analysis was conducted on an Orbitrap Astral mass spectrometer coupled to a Vanquish Neo UHPLC system. Peptides were separated on a 50 cm µPAC™ Neo HPLC column using a gradient of 0.1% formic acid in water (A) and 0.1% formic acid in 80% acetonitrile (B) at 2.2 μL/min, with an 8 min elution. DIA acquisition included 380–980 m/z full MS scans and 150–2000 m/z DIA MS/MS scans, with a cycle time of 0.6 s and normalized collision energy of 25.DIA data were analyzed using DIA-NN 1.8.1 against the UniProtKB Swiss-Prot (*Homo sapiens*) database. Trypsin was set as the digestion enzyme with one allowed missed cleavage. Cysteine carbamidomethylation was specified as a fixed modification, while protein N-terminal acetylation and methionine oxidation were set as variable modifications. Peptides of 7–30 amino acids with charges 1–4 and fragment ions in the 150–2000 m/z range were considered. Peptide-spectrum matches and protein identifications were filtered at <1% FDR before export.

### Real-time quantitative PCR

RT-qPCR was employed to assess *Helicobacter pylori* (HP) colonization and the mRNA expression of PXR downstream targets in mouse gastric tissues. Total RNA was extracted using TRIzol (Invitrogen) and quantified with a NanoDrop 2000 (A260/A280 = 1.8–2.0). One microgram of RNA was reverse-transcribed into cDNA using the PrimeScript™ RT reagent kit (TaKaRa). RT-qPCR was performed with TB Green Premix Ex Taq™ II (TaKaRa) on a real-time PCR system under the following cycling conditions: 95 °C for 30 s pre-denaturation, followed by 40 cycles of 95 °C for 5 s and 60 °C for 34 s, with subsequent melting curve analysis to confirm specificity.HP colonization was quantified via 16S rRNA and urease A (ureA) genes, normalized to Gapdh. PXR activation was evaluated by measuring its canonical targets, Cyp3a11 and Abcb1a. Additionally, expression of Wnt/β-catenin pathway genes (Axin2, Ctnnb1) and EMT markers (Snai2, Zeb1) was assessed, all normalized to Gapdh. Reactions were performed in triplicate, and relative expression was calculated using the 2^−^ΔΔCt method.

### Immunofluorescence staining

After embedding in OCT compound, gastric tissues were stored at −80 °C and sectioned into 10 μm frozen slices. Sections were air-dried, fixed with 4% paraformaldehyde for 15 min, permeabilized with 0.1% Triton X-100 for 15 min, and blocked with 5% donkey serum for 1 h at room temperature. Primary antibodies against E-cadherin (1:250, Proteintech, #20874-1-AP), N-cadherin (1:250, Affinity, #AF5239), β-catenin (1:100, Proteintech, #51067-2-AP), and PXR (1:200, Abcam, ab192579) were incubated overnight at 4 °C. After washing with PBST, sections were incubated with Alexa Fluor 488–conjugated secondary antibody (1:200, Invitrogen) for 1 h at room temperature in the dark. Nuclei were counterstained with Hoechst 33342 (1:1,000) for 10 min. Images were captured using a Nikon Eclipse Ti fluorescence microscope.For cellular immunofluorescence, cells were seeded on coverslips and grown to 70%–80% confluence. Following treatment, cells were fixed, permeabilized, and blocked as described above, then incubated with the same primary and secondary antibodies. β-catenin nuclear translocation and PXR subcellular localization were specifically evaluated. All images were acquired under identical exposure conditions, and fluorescence intensity was quantified using ImageJ software.

### Statistical analysis

Data are presented as mean ± standard deviation (SD) or mean ± standard error of the mean (SEM). Statistical analyses were performed using R software. For two-group comparisons, normally distributed data were analyzed by independent-samples t-test, and non-normally distributed data by the Mann–Whitney U test. For multiple-group comparisons, one-way ANOVA was used, followed by Tukey’s HSD or Dunnett’s *post hoc* tests. Correlations were assessed using Spearman’s coefficient. P values <0.05 were considered statistically significant, indicated as *P < 0.05, **P < 0.01, ***P < 0.001, and ****P < 0.0001. During initial proteomics and metabolomics screening, raw P values were used for exploratory analyses without multiple hypothesis correction (e.g., FDR or BH adjustment) to avoid overly conservative results that could obscure potential biological signals in limited sample sizes.

## Results

### HP-associated gastric cancer shows PXR downregulation, Wnt/β-catenin activation, and EMT

**FIGURE 1:**
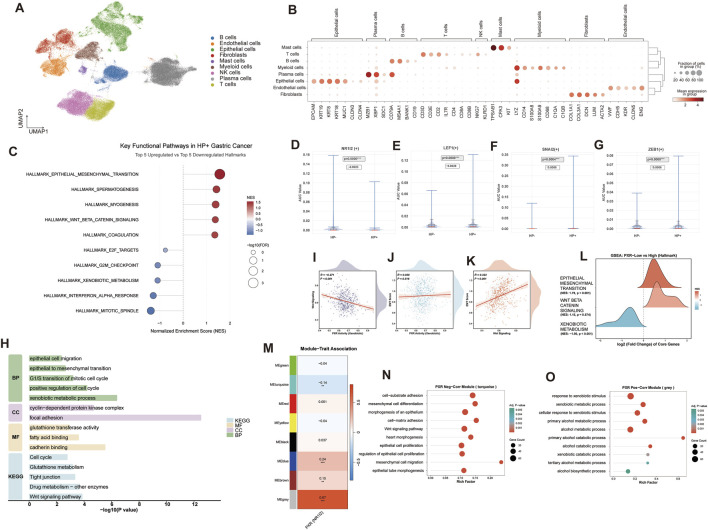
Analysis workflow of the PXR–Wnt/β-catenin–EMT axis in gastric epithelial cells under *Helicobacter pylori* (HP) infection. Single-cell RNA-seq of gastric cancer tissues identified major cell populations visualized by UMAP and annotated using canonical marker genes **(A,B)**. GSEA compared pathway enrichment between HP^+^ and HP^−^ epithelial cells **(C)**. pySCENIC assessed transcription factor activity, including NR1I2 (PXR) and Wnt/EMT-related factors LEF1, SNAI2, and ZEB1 **(D–G)**. Virtual knockout of NR1I2 using scTenifoldKnk evaluated effects on EMT, Wnt signaling, and associated pathways **(H)**. Correlation analyses in the TCGA-STAD cohort examined the relationship between PXR activity and Wnt/EMT pathway scores **(I–K)**, with GSEA performed on samples stratified by PXR activity **(L)**. WGCNA identified PXR-associated gene modules and their functional enrichment, including modules related to Wnt signaling, cell adhesion, and xenobiotic metabolism **(M–O)**.

Publicly available single-cell transcriptomic data, comprising *Helicobacter* pylori–positive (HP^+^) and HP-negative (HP^−^) gastric cancer samples, were analyzed. Data processing, including quality control, normalization, and dimensionality reduction, was performed using Seurat, and cell populations were annotated based on canonical marker genes. UMAP visualization revealed heterogeneous tumor tissues comprising T cells, B cells, NK cells, plasma cells, myeloid cells, mast cells, epithelial cells, endothelial cells, and fibroblasts (Figures 1A,B).

To explore the impact of HP infection on the molecular phenotype of gastric epithelial cells, GSEA was conducted comparing HP^+^ and HP^−^ epithelial cells. HP^+^ epithelial cells exhibited significant enrichment of EMT and Wnt/β-catenin signaling pathways, whereas HP^−^ epithelial cells were enriched for HALLMARK_XENOBIOTIC_METABOLISM, reflecting xenobiotic metabolism and detoxification programs centered on the PXR (Figure 1C). These results suggest that HP infection suppresses PXR-related metabolic activity while promoting Wnt signaling and EMT programs in epithelial cells.

Additionally, transcription factor activity was assessed using pySCENIC to construct single-cell gene regulatory networks. NR1I2 (PXR) activity was significantly reduced in HP^+^ epithelial cells, whereas Wnt/β-catenin regulator LEF1 and EMT core factors SNAI2 and ZEB1 exhibited marked upregulation (Figures 1D–G).

### Virtual knockout and clinical cohort analysis reveal a causal link between PXR and Wnt/EMT

To investigate whether reduced PXR expression functionally drives Wnt activation and the EMT phenotype, a virtual knockout of NR1I2 was performed using scTenifoldKnk, simulating the PXR-suppressed state observed under HP infection. Enrichment analysis of the differentially expressed genes following virtual knockout (Figure 1H) revealed extensive transcriptional network remodeling. At the biological process level, genes were primarily enriched in EMT, epithelial cell migration, and cell cycle–related processes. KEGG pathway analysis indicated significant enrichment of Wnt signaling and tight junction pathways, while classical PXR-related pathways, including xenobiotic and glutathione metabolism, were concomitantly affected.

To assess clinical relevance, these findings were extended to the TCGA-STAD cohort. Correlation analyses demonstrated a significant negative association between PXR activity and Wnt signaling scores, whereas Wnt pathway scores positively correlated with EMT scores (Figures 1I–K). Stratification into high- and low-PXR activity groups for GSEA showed that low-PXR samples exhibited significant enrichment of EMT, a trend toward Wnt/β-catenin activation, and marked downregulation of xenobiotic metabolism pathways (Figure 1L).

Furthermore, WGCNA was applied to define PXR-associated gene networks (Figure 1M). Modules negatively correlated with PXR expression were enriched in Wnt signaling, mesenchymal differentiation and migration, and cell–matrix adhesion pathways (Figure 1N), whereas modules positively correlated with PXR were predominantly associated with classical PXR functions, including xenobiotic metabolism (Figure 1O).

### Molecular docking and dynamics simulation confirm stable binding of Patchouli alcohol to PXR

**FIGURE 2:**
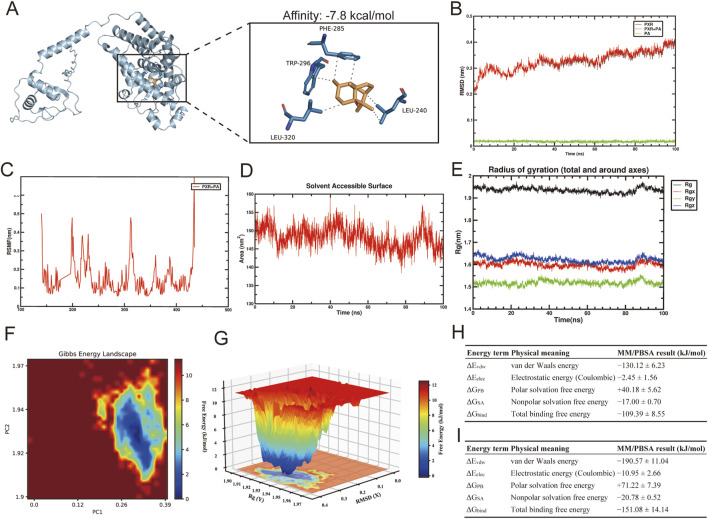
Molecular docking and molecular dynamics (MD) analysis of PA binding to PXR. PA docking within the ligand-binding domain (LBD) of PXR, highlighting hydrophobic pocket interactions with key residues LEU-209, VAL-211, PHE-288, TRP-299, and TYR-306 **(A)**. MD simulations over 100 ns assessed structural stability via RMSD of the PXR backbone, PA–PXR complex, and ligand **(B)**, and residue flexibility via RMSF **(C)**. Complex stability was further evaluated by solvent-accessible surface area (SASA) dynamics **(D)** and radius of gyration (Rg) with its three-dimensional components **(E)**. Gibbs free energy landscape (FEL) analysis based on PC1, PC2, RMSD, and Rg **(F,G)**. MM/PBSA analysis demonstrated that PA exhibits favorable binding free energy with PXR, with binding affinity comparable to the positive control PCN **(H,I)**.

Molecular docking simulations revealed the structural basis of PA–PXR interaction. PA stably occupies the hydrophobic pocket of the PXR ligand-binding domain (LBD) with a binding energy of −7.8 kcal/mol, and complex stability is primarily driven by hydrophobic interactions with key residues LEU-209, VAL-211, PHE-288, TRP-299, and TYR-306 (Figure 2A).

To evaluate complex stability in an aqueous environment, a 100-ns molecular dynamics (MD) simulation was performed. The backbone RMSD of PXR and the PA–PXR complex stabilized after initial adjustments within the first 20 ns (∼0.4 nm, Figure 2B), while PA remained tightly bound within the active site, exhibiting minimal RMSD (∼0.02 nm). RMSF analysis indicated low flexibility (<0.15 nm) for pocket residues, except at the N- and C-terminal regions, suggesting that PA reinforces the structural stability of the binding pocket (Figure 2C). SASA and radius of gyration analyses confirmed that both the hydrophobic core and the overall protein conformation remained stable, with no unfolding or expansion observed (Figures 2D,E). The free energy landscape (FEL) revealed a single, well-defined energy minimum, indicating that the PA–PXR complex adopts a thermodynamically stable conformation (Figures 2F,G).

MM/PBSA calculations showed that the total binding free energy of PA with PXR was −109.39 ± 8.55 kJ/mol (Figure 2H), compared with −151.08 ± 14.14 kJ/mol for the positive control PCN (Figure 2I), indicating that PA exhibits moderate to strong binding affinity, comparable to PCN at the theoretical level.

### Macroscopic evaluation of gastric tissues

**FIGURE 3:**
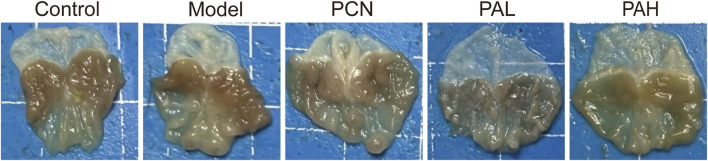
Gross morphology of gastric tissues in each experimental group. The control group exhibited smooth gastric mucosa with well-defined folds. In contrast, the model group (MNU + HP) showed thickened folds and focal elevated lesions. Treatment with PCN and PA ameliorated these morphological abnormalities to varying extents. Notably, PA demonstrated a dose-dependent protective effect, indicating attenuation of MNU- and HP-induced gastric pathological damage.

At the end of the animal experiments, the gastric tissues from each group of mice were examined macroscopically (Figure 3). In the control group, the gastric mucosa was smooth, with clear folds and no obvious tumor-like lesions. In the model group (MNU + HP), the gastric mucosal folds were thickened, with localized mild elevations and gastric wall thickening, indicating that MNU combined with HP infection successfully induced tumor-like pathological changes in the gastric mucosa. In the PCN group, gastric lesions were alleviated compared with the model group, with fewer mucosal nodules. In the low-dose PA (PAL) group, the smoothness of the gastric mucosa was significantly improved, and nodular lesions were markedly reduced. The high-dose PA (PAH) group showed even more pronounced effects, with gastric mucosal morphology approaching normal, demonstrating a dose-dependent inhibitory effect of PA on HP plus MNU-induced gastric mucosal lesions. These results indicate that MNU combined with HP can successfully establish a gastric mucosal lesion model, and PA intervention can effectively alleviate gross pathological damage.

### PA modulates bile acid–lipid metabolism and suppresses Wnt/EMT signaling

**FIGURE 4:**
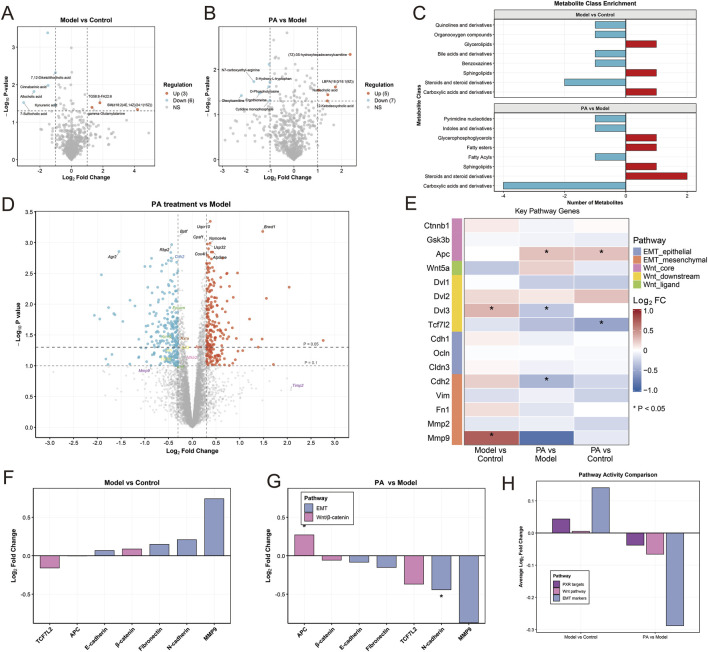
Integrated metabolomic and proteomic analysis. **(A–C)** Untargeted metabolomics. **(A)** Volcano plot of differential metabolites between Model and Control groups (P < 0.05, |log2FC| > 1). **(B)** Volcano plot of differential metabolites between PA and Model groups. **(C)** Metabolite class enrichment analysis: top, Model vs. Control; bottom, PA vs. Model. **(D–G)** Proteomics. **(D)** Volcano plot of differential proteins between PA and Model groups. **(E)** Heatmap of key pathway proteins, highlighting Wnt/β-catenin and EMT-related protein expression. **(F,G)** Quantitative bar plots of selected proteins comparing expression trends across groups. **(H)** Comparative pathway activity analysis integrating metabolomic and proteomic data.

Untargeted metabolomic analysis demonstrated that, compared with the Control group, nine metabolites were significantly altered in the Model group (P < 0.05, |log_2_FC| > 1), including three upregulated and six downregulated metabolites (Figure 4A). Notably, bile acid–related metabolites—including *7,12-diketolithocholic acid, allocholic acid, and 7-sulfocholic acid*—constituted a major proportion of the downregulated metabolites. In addition, several lipid metabolites, such as *TDBS-PA22S and SM(d18:2/24:1)(12Z)*, were markedly reduced. Given that bile acids function as endogenous ligands of PXR, their pronounced depletion suggests that HP infection combined with MNU exposure may compromise ligand-dependent PXR activation. In contrast, PA treatment induced significant alterations in 12 metabolites relative to the Model group (P < 0.05, |log_2_FC| > 1), comprising five upregulated and seven downregulated metabolites (Figure 4B). The upregulated metabolites were predominantly lipid-associated, including (7Z)-3S-hydroxyhexadecenoylcarnitine and *LSM(18:1/18:1)(9Z)*, whereas the downregulated metabolites were mainly related to amino acid and nucleotide metabolism. Metabolite class enrichment analysis further revealed that PA treatment significantly increased steroids and their derivatives (including bile acids), sphingolipids, fatty acyls, and glycerophosphoglycerols, indicating a broad restoration of lipid metabolic homeostasis (Figure 4C).

Proteomic profiling revealed that PA profoundly reshaped the protein expression landscape (Figure 4D). Heatmap analysis showed that, compared with the Control group, the Model group exhibited marked upregulation of core Wnt signaling components (including Ctnnb1, Dvl2, and prominently Dvl3), along with elevated expression of mesenchymal EMT markers (Cdh2, Fn1, and notably Mmp9), indicating activation of the Wnt/β-catenin pathway and induction of EMT following HP infection combined with MNU exposure. Importantly, PA treatment effectively reversed these pathological alterations. Within the Wnt pathway, PA significantly reduced the expression of Dvl3 (P < 0.05) and concomitantly downregulated Tcf7l2. In parallel, PA markedly suppressed the mesenchymal markers Cdh2 (N-cadherin) and Mmp9 (P < 0.05), while significantly restoring the expression of the Wnt pathway negative regulator and epithelial integrity–associated protein APC (P < 0.05) (Figure 4E). Quantitative analyses further confirmed that PA treatment significantly increased APC levels and concomitantly reduced N-cadherin expression (P < 0.05) (Figures 4F,G). Integrated pathway activity analysis demonstrated that Wnt signaling and EMT programs were globally activated in the Model group but were markedly suppressed following PA intervention (Figure 4H).

### RT-qPCR validation of PXR targets, Wnt/β-catenin pathway, and EMT markers

**FIGURE 5:**
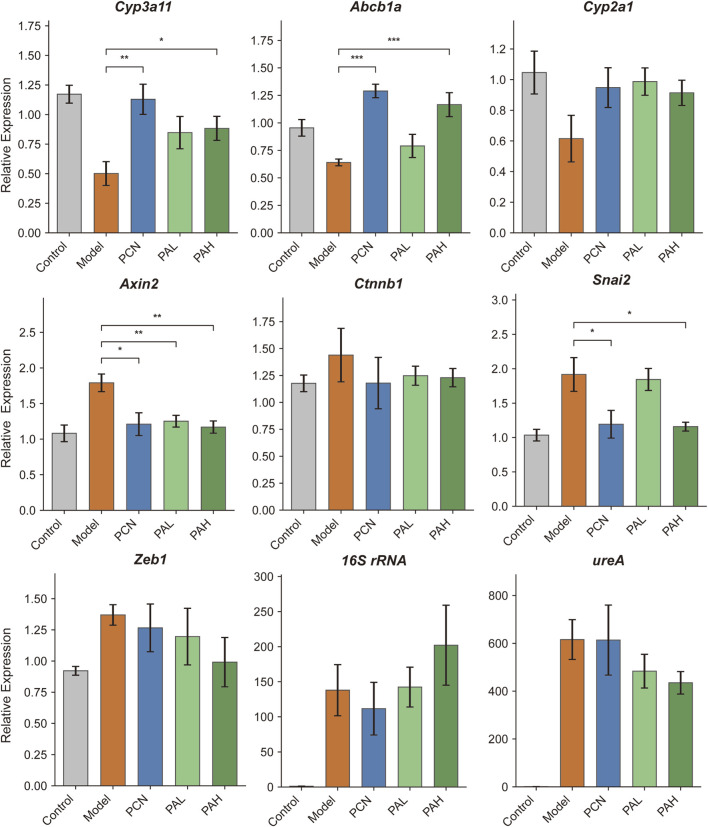
RT-qPCR analysis of PXR target genes, Wnt/β-catenin pathway genes, EMT markers, and HP colonization in mouse gastric tissues.

RT-qPCR analysis revealed that, compared with controls, the model group (MNU + HP) exhibited significant downregulation of the classical PXR targets Cyp3a11 and Abcb1a in mouse gastric tissues (P < 0.05), indicating suppressed PXR transcriptional activity following combined HP infection and MNU treatment. Treatment with the PXR agonist PCN restored Cyp3a11 and Abcb1a expression (P < 0.01), confirming effective PXR activation. Both low-dose (PAL) and high-dose (PAH) PA treatments produced similar upregulation trends, suggesting that PA can activate PXR. Cyp2a1 expression remained unchanged across groups, indicating gene-specific effects. For the Wnt/β-catenin pathway, Axin2 mRNA was significantly elevated in the model group, whereas PCN, PAL, and PAH significantly reduced its expression (P < 0.05–0.01), suggesting that PXR activation suppresses Wnt transcriptional output. Ctnnb1 (β-catenin) expression showed no significant differences, consistent with protein-level data, indicating that regulation occurs primarily at the transcriptional level. Regarding EMT markers, Snai2 mRNA was significantly increased in the model group and was markedly reduced by PCN and PAH treatments (P < 0.05), with PAL showing a downward trend. Zeb1 expression also tended to decrease following PCN and PA treatments but did not reach statistical significance. Analysis of HP colonization indicated that 16S rRNA and ureA levels were significantly higher in the model group. Intervention with PCN, PAL, or PAH produced slight, non-significant reductions, suggesting that PA primarily modulates host signaling pathways rather than directly affecting bacterial colonization (Figure 5).

### Immunofluorescence validation of PXR–Wnt/β-catenin axis and EMT

**FIGURE 6:**
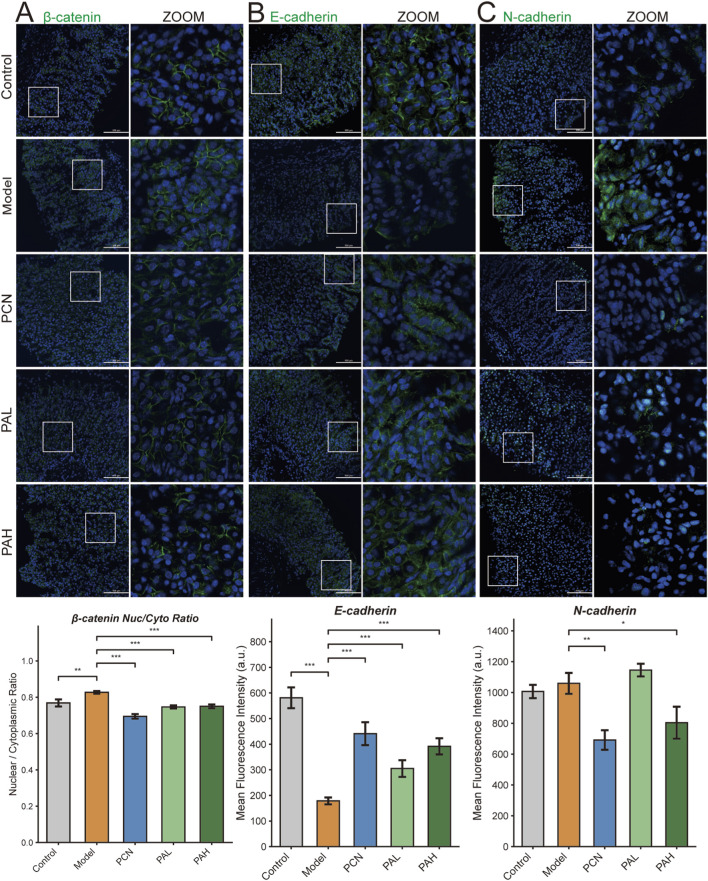
Immunofluorescence analysis of the effects of PA on Wnt signaling and EMT phenotypes in gastric tissues. Representative immunofluorescence staining of β-catenin with nuclear counterstaining by Hoechst 33342 **(A)**, the epithelial marker E-cadherin **(B)**, and the mesenchymal marker N-cadherin **(C)**. Magnified views of selected areas (ZOOM) are shown for each panel.

To validate proteomics findings at the tissue level, gastric tissues were subjected to immunofluorescence staining for EMT markers. Quantitative analysis of the β-catenin nuclear-to-cytoplasmic ratio (Nuc/Cyto) revealed a significant increase in the model group compared with controls (P < 0.01), indicating that HP combined with MNU promotes β-catenin nuclear translocation and activates Wnt transcriptional programs. PCN, PAL, and PAH treatments significantly reduced the Nuc/Cyto ratio (P < 0.001), demonstrating that PXR activation inhibits β-catenin nuclear accumulation (Figure 6A). E-cadherin fluorescence intensity was markedly decreased in the model group (P < 0.001), reflecting loss of epithelial characteristics, whereas PCN and PA treatments restored expression in a dose-dependent manner (P < 0.001) (Figure 6B). N-cadherin showed an increasing trend in the model group, was significantly reduced by PCN (P < 0.01), slightly decreased in PAL, and significantly suppressed in PAH (P < 0.05), also in a dose-dependent manner (Figure 6C). Overall, tissue immunofluorescence results were consistent with proteomics data, indicating that PA reverses the EMT phenotype of HP-associated gastric cancer by activating PXR, inhibiting β-catenin nuclear translocation, restoring E-cadherin, and downregulating N-cadherin.

**FIGURE 7:**
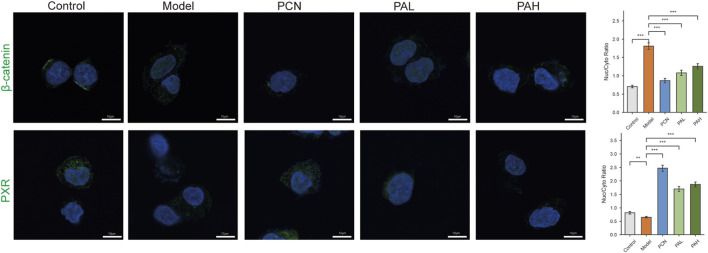
Immunofluorescence analysis and nuclear-to-cytoplasmic ratio quantification of β-catenin and PXR in HP-infected cells.

At the cellular level, PA’s regulation of the PXR–Wnt axis was further confirmed. In HP-infected cells, β-catenin Nuc/Cyto ratio was significantly increased in the model group (P < 0.001), indicating enhanced nuclear translocation and Wnt activation. PCN, PAL, and PAH treatments markedly reduced this ratio (P < 0.001), confirming that PXR activation suppresses β-catenin nuclear accumulation (Figure 7). PXR nuclear-to-cytoplasmic ratio was significantly decreased in the model group (P < 0.01), reflecting inhibition of PXR nuclear translocation by HP infection. Both PCN and PA treatments significantly restored PXR nuclear localization (P < 0.001), with comparable effects for low and high PA doses.

## Discussion

HP infection remains a predominant etiological factor in gastric carcinogenesis, accounting for approximately 90% of non-cardia gastric cancers (Salvatori et al., 2023). Although widespread implementation of HP eradication therapy has led to a partial decline in gastric cancer incidence, a considerable proportion of patients continue to progress to advanced disease stages. This underscores a critical unmet need for therapeutic strategies that specifically target HP-associated molecular dysregulation. In the present study, we systematically investigated the antitumor potential of PA in HP-associated gastric cancer and demonstrated that PA exerts its inhibitory effects, at least in part, through modulation of the PXR–Wnt/β-catenin–EMT signaling axis.

Single-cell transcriptomic profiling revealed that *Helicobacter pylori*–positive gastric cancer epithelial cells display a distinct molecular phenotype characterized by attenuated PXR activity, concomitant activation of Wnt/β-catenin signaling, and induction of the EMT program. GSEA demonstrated significant suppression of the HALLMARK_XENOBIOTIC_METABOLISM pathway—representative of PXR-mediated xenobiotic detoxification—in HP^+^ epithelial cells, whereas EMT-related and Wnt/β-catenin signaling pathways were robustly enriched. These observations are consistent with prior evidence that HP infection facilitates gastric tumorigenesis through extensive reprogramming of host signaling networks (Yong et al., 2015; He et al., 2025). Notably, however, the involvement of PXR in HP-associated gastric cancer has not been previously elucidated.

PXR is a nuclear receptor traditionally recognized as a central regulator of xenobiotic metabolism and detoxification (Lv et al., 2022; Dutta et al., 2022). Emerging evidence indicates that PXR functions extend beyond metabolic regulation, encompassing roles in inflammation, cell proliferation, and tumorigenesis (Robbins and Chen, 2014; Sun et al., 2022). Interestingly, PXR exhibits context-dependent, bidirectional effects in cancer: it promotes tumor progression in colorectal and ovarian cancers (Gupta et al., 2008; Dong et al., 2017), while exerting tumor-suppressive functions in prostate and breast cancers (Fujimura et al., 2012; Verma et al., 2009). In the present study, PXR was found to act as a tumor suppressor in HP-associated gastric cancer. Consistently, pySCENIC analysis revealed a marked reduction in NR1I2 regulatory activity in HP^+^ epithelial cells, accompanied by upregulation of key Wnt/EMT transcription factors, including LEF1, SNAI2, and ZEB1, suggesting that PXR downregulation contributes to Wnt/EMT pathway activation.

To assess the causal link between PXR loss and Wnt/EMT activation, we performed a virtual knockout of NR1I2 using scTenifoldKnk, which models gene perturbation at the single-cell level (Osorio et al., 2022). PXR virtual knockout induced substantial remodeling of the transcriptional network, with differentially expressed genes enriched in EMT, epithelial cell migration, Wnt signaling, and tight junction–associated processes, alongside concurrent downregulation of xenobiotic metabolism pathways, confirming the specificity of the perturbation. These results provide functional evidence supporting the mechanism whereby HP infection downregulates PXR, leading to activation of the Wnt/EMT pathway.

The single-cell observations were further corroborated in the TCGA-STAD cohort. Correlation analysis revealed a significant negative association between PXR activity and Wnt pathway scores, whereas Wnt and EMT scores were positively correlated. GSEA stratified by PXR activity demonstrated that low PXR expression was linked to EMT enrichment and Wnt/β-catenin pathway activation. WGCNA identified gene modules inversely correlated with PXR expression, which were enriched in Wnt signaling, mesenchymal differentiation, and cell–matrix adhesion pathways. Collectively, these results extend the single-cell findings to a clinical cohort, indicating that dysregulation of the PXR–Wnt–EMT axis is a characteristic feature of gastric cancer progression.

PA, a tricyclic sesquiterpene derived from *Pogostemon cablin*, possesses anti-inflammatory, antimicrobial, and antitumor properties (Lian et al., 2018; Song et al., 2021; Xian et al., 2011).Its role and molecular targets in gastric cancer, however, remain largely unexplored. Molecular docking analysis indicated that PA fits within the ligand-binding domain of PXR, forming stable hydrophobic interactions with key residues that constitute the conserved hydrophobic core of the LBD, corresponding to the binding sites of established PXR ligands such as rifampicin and SR12813 (Watkins et al., 2001; Chrencik et al., 2005). Subsequent 100-ns molecular dynamics simulations demonstrated that the PA–PXR complex stabilized after ∼20 ns, with the protein backbone RMSD converging at ∼0.4 nm. RMSF analysis revealed minimal fluctuations (<0.15 nm) within the binding pocket, indicating structural stability. MM/PBSA calculations estimated a binding free energy of −109.39 ± 8.55 kJ/mol, consistent with high-affinity interactions comparable to known PXR agonists (Genheden and Ryde, 2015; Huber et al., 2021). These results provide a theoretical basis for PA as a potential PXR activator in gastric cancer.

Metabolomic analysis revealed that HP infection combined with MNU treatment significantly reduced bile acid–related metabolites, including 7,12-diketolithocholic acid, allocholic acid, and 7-sulfocholic acid, whereas PA treatment partially restored their levels. Bile acids serve as key endogenous ligands for PXR, critically mediating its physiological activation (Dutta et al., 2022; Staudinger et al., 2001; Goodwin et al., 2003). Based on these observations, we propose a “bile acid–PXR–Wnt” regulatory model: HP infection perturbs bile acid homeostasis, attenuating ligand-dependent PXR activation and thereby relieving its inhibitory effect on Wnt/β-catenin signaling, which promotes EMT and tumor progression. Conversely, PA, as an exogenous PXR agonist, can bypass the deficit of endogenous ligands to directly activate PXR, suppressing Wnt signaling and the EMT program.

Notably, our findings suggest that PA activates PXR through two complementary mechanisms. First, molecular docking and molecular dynamics simulations indicate that PA directly binds to the PXR ligand-binding domain with high affinity (ΔG = −109.39 ± 8.55 kJ/mol) and forms a stable complex, supporting its potential agonistic activity. Second, metabolomics analysis showed that PA restored bile acid levels depleted following HP infection, implying indirect PXR activation via improvement of the gastrointestinal metabolic milieu and replenishment of endogenous ligands. These mechanisms are not mutually exclusive but likely represent distinct aspects of PA’s multi-target regulation. Direct binding may initiate rapid PXR activation, whereas restoration of bile acid homeostasis may sustain its activity over time. Moreover, metabolic reprogramming induced by PA may exert additional antitumor effects independent of PXR, including attenuation of oxidative stress, enhancement of epithelial barrier integrity, and modulation of the inflammatory microenvironment (Song et al., 2021; Lee et al., 2023). Thus, characterizing PA solely as a “PXR agonist” or a “metabolic regulator” may underestimate its functional scope; rather, PA appears to coordinately activate PXR while broadly modulating metabolic and oncogenic signaling pathways involved in gastric carcinogenesis. However, direct physical interaction between PA and PXR has not yet been validated by *in vitro* reporter assays or binding studies, nor have time-course experiments been conducted to distinguish the relative contributions of direct versus indirect activation. Furthermore, the relationship between the effective *in vivo* concentration of PA and the predicted binding affinity requires further experimental confirmation.

Proteomic analysis showed that HP infection combined with MNU treatment markedly activated the Wnt/β-catenin pathway and induced EMT, evidenced by upregulation of core Wnt proteins (Dvl2, Dvl3, Ctnnb1) and mesenchymal markers (N-cadherin/Cdh2, Fibronectin/Fn1, MMP9) in the Model group, along with downregulation of the Wnt negative regulator APC and epithelial maintenance proteins, reflecting loss of the epithelial phenotype. Following PA treatment, Dvl3 and Tcf7l2 levels decreased, N-cadherin and MMP9 were significantly downregulated, and APC expression was restored, indicating that PA reverses EMT by inhibiting Wnt signaling and restoring epithelial protein expression. Immunofluorescence staining of tissue further validated these findings: PA reduced β-catenin accumulation, partially restored E-cadherin expression, and dose-dependently suppressed N-cadherin (PAH > PAL). The classical PXR agonist PCN exhibited similar effects, supporting that PA inhibits the Wnt/EMT pathway via PXR activation.

RT-qPCR analysis further validated the PXR–Wnt–EMT regulatory axis at the transcriptional level. In the model group, the canonical PXR target genes Cyp3a11 and Abcb1a were significantly downregulated, whereas PA and PCN treatment restored their expression, confirming activation of PXR transcriptional activity by PA. These findings are consistent with metabolomic evidence of bile acid restoration and molecular docking results supporting PA–PXR interaction. The Wnt target gene Axin2 was markedly upregulated in the model group but significantly reduced following PA intervention, indicating suppression of β-catenin–dependent transcription. This result aligns with the proteomic downregulation of Dvl3 and Tcf7l2, as well as decreased β-catenin nuclear translocation observed by immunofluorescence. Similarly, the EMT-associated transcription factor Snai2 was significantly elevated in the model group and downregulated by PA and PCN treatment. Together with pySCENIC predictions from single-cell analysis and the corresponding changes in EMT protein markers (decreased N-cadherin and increased E-cadherin), these data provide multi-level validation—spanning transcriptional, proteomic, and phenotypic evidence—supporting the PXR–Wnt–EMT regulatory axis.

A critical objective of this study was to distinguish the antimicrobial activity of PA from its regulatory effects on host signaling pathways. Given previous reports of PA’s antibacterial properties (Xu et al., 2017; Zhong et al., 2021), it was essential to determine whether its inhibition of the Wnt/EMT pathway results from direct activation of host PXR or secondary effects due to HP eradication. RT-qPCR analysis of HP colonization in mouse gastric tissues showed that 16S rRNA and ureA expression were significantly elevated in the model group compared with controls (P < 0.001), confirming successful infection. Although PA-treated groups (PAL and PAH) exhibited a downward trend in HP colonization, the differences were not statistically significant (P > 0.05). No significant change was observed in the PCN group. These results indicate that the therapeutic effects of PA and PCN are unlikely to be primarily mediated by direct antibacterial activity, but instead are more plausibly attributed to modulation of host signaling pathways.

Further evidence indicates that PA’s effects are primarily mediated through the PXR pathway rather than direct antibacterial activity. First, despite no significant changes in HP colonization, PA markedly improved bile acid metabolism (metabolomics), activated PXR target genes (Cyp3a11, Abcb1a; RT-qPCR), and suppressed Wnt/EMT pathway proteins (proteomics and immunofluorescence), demonstrating effects independent of bacterial load. Second, PCN, a classical PXR agonist lacking antibacterial activity, similarly inhibited Wnt/EMT signaling without affecting HP colonization, serving as a positive control for PXR-dependent effects. Third, if PA’s action were primarily due to bacterial clearance, HP colonization and Wnt/EMT activation would be expected to correlate in a dose-dependent manner, which was not observed. Collectively, these results indicate that PA exerts its antitumor effects mainly via activation of the host PXR–Wnt/EMT signaling axis. Any minor antibacterial activity, such as the modest downward trend in HP colonization, is unlikely to be the primary mechanism.

This study has several limitations. First, the regulatory effect of PA on the PXR–Wnt–EMT axis has not been directly validated *in vitro* gastric cancer cell lines. Second, although virtual knockout analysis suggested that PXR deficiency can activate the Wnt/EMT pathway, experimental evidence from PXR knockout or overexpression models is lacking to confirm the PXR-dependence of PA’s effects. Third, this study relied on publicly available single-cell datasets and the TCGA-STAD cohort, and their clinical relevance in independent HP-associated gastric cancer cohorts has not yet been validated. Fourth, the relative contributions of the direct mechanism of PXR activation (ligand binding) versus the indirect mechanism (bile acid restoration) have not been clearly distinguished through systematic time-course or mechanistic experiments, limiting precise understanding of PA’s mode of action. Fifth, the animal experiments were conducted exclusively in male C57BL/6 mice without sex-based comparisons; given that sex hormones may influence gastric carcinogenesis and the expression of drug-metabolizing enzymes, the applicability of these findings to female mice and female patients requires further investigation.

## Conclusion

In summary, this study identifies a novel mechanism by which PA suppresses HP-associated gastric cancer through direct and indirect activation of PXR, leading to inhibition of the Wnt/β-catenin pathway and EMT. PA activates PXR both by directly binding its ligand-binding domain and by restoring bile acid metabolism depleted by HP infection. These multi-target regulatory effects position PA as a promising therapeutic candidate for HP-associated gastric cancer. The findings provide a foundation for future clinical translation and interventional studies, while further investigation is warranted to fully elucidate the precise mechanisms of PA action.

## Data Availability

The datasets presented in this study can be found in the Zenodo repository. The data are available at https://zenodo.org/records/19235060.
